# Long Non-Coding RNAs in Insects

**DOI:** 10.3390/ani11041118

**Published:** 2021-04-14

**Authors:** Chhavi Choudhary, Shivasmi Sharma, Keshav Kumar Meghwanshi, Smit Patel, Prachi Mehta, Nidhi Shukla, Duy Ngoc Do, Subhash Rajpurohit, Prashanth Suravajhala, Jayendra Nath Shukla

**Affiliations:** 1 Department of Biotechnology, School of Life Sciences, Central University of Rajasthan, Bandarsindari, Ajmer 305801, India; 2019phdbt004@curaj.ac.in (C.C.); 2019phdbt008@curaj.ac.in (K.K.M.); 2 Department of Biotechnology, Amity University Jaipur, Jaipur 303002, India; shivasmisharma646@gmail.com (S.S.); patelsmit11103@gmail.com (S.P.); 3 Division of Biological & Life Sciences, School of Arts and Sciences, Ahmedabad University, Gujarat 380009, India; prachi.um.imsc15@ahduni.edu.in (P.M.); subhash.rajpurohit@ahduni.edu.in (S.R.); 4 Department of Biotechnology and Bioinformatics, Birla Institute of Scientific Research, Jaipur 302001, India; nidhiaaidu@gmail.com; 5 Institute of Research and Development, Duy Tan University, Danang 550000, Vietnam; Duy.Do@dal.ca; 6 Bioclues.org, Vivekananda Nagar, Kukatpally, Hyderabad, Telangana 500072, India

**Keywords:** non-coding RNA, insects, LncRNAs, regulatory functions, development

## Abstract

**Simple Summary:**

Long-non-coding RNAs (lncRNAs) are transcripts of more than 200 nucleotides, which lack protein-coding potential. LncRNAs have been well characterized in many organisms, but their functions in insects have not been well deciphered. Advancement of high-throughput technologies has enabled the sequencing of genomes and transcriptomes of several insects, which has led to the identification of many important lncRNAs in insects. Characterization of lncRNAs and their regulatory roles in insects may provide insights of novel pest control strategies. Through this comprehensive review, we present an overview of insect lncRNAs, their identification as well as their function in insects of different orders. Toward the end of the review, we highlight the role of lncRNAs in insect developmental processes and discuss the future prospects of lncRNAs in insects.

**Abstract:**

Only a small subset of all the transcribed RNAs are used as a template for protein translation, whereas RNA molecules that are not translated play a very important role as regulatory non-coding RNAs (ncRNAs). Besides traditionally known RNAs (ribosomal and transfer RNAs), ncRNAs also include small non-coding RNAs (sncRNAs) and long non-coding RNAs (lncRNAs). The lncRNAs, which were initially thought to be junk, have gained a great deal attention because of their regulatory roles in diverse biological processes in animals and plants. Insects are the most abundant and diverse group of animals on this planet. Recent studies have demonstrated the role of lncRNAs in almost all aspects of insect development, reproduction, and genetic plasticity. In this review, we describe the function and molecular mechanisms of the mode of action of different insect lncRNAs discovered up to date.

## 1. Introduction

**Figure 1:**
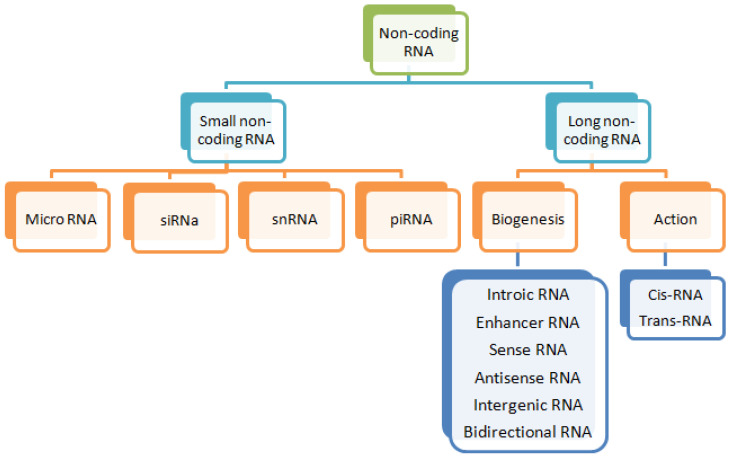
Classification of non-coding RNA. Non-coding RNA can be divided into two groups (small and long non-coding RNA) based on their size. Small non-coding RNA can be further divided into miRNA, piRNA, siRNA, etc. Long non-coding RNA can be subdivided into sense, anti-sense, intronic, intergenic, cis-, and trans-RNA based on their biogenesis and mechanism of action (modified from Dahariya et al. [8]).

Eukaryotic genomes are known to produce a huge array of RNA molecules differing in their size, abundance, and protein coding ability [1]. Only 2% of RNA is translated to make proteins; other RNA sequences that do not code for any protein are called non-coding RNA (ncRNAs) [2]. While the proteins are considered as the major trans-acting regulators, they are limited by the organizational complexity in forming a regulatory network. This is partially explained by the alternative splicing in the pre-mRNA of protein-coding genes and by the post-translational modification of proteins [3]. A paradigm shift has occurred in recent years with the identification and functional characterization of an increasing number of ncRNAs. The knowledge of ncRNAs has gained a huge momentum with the discovery of RNA interference (RNAi) and the role of ncRNAs in gene silencing phenomena [4]. This has led to the identification of various types of small ncRNAs, which guide the protein complex to the target mRNA to influence its translation. The ncRNAs include ribosomal RNA (rRNA), transfer RNA (tRNA), small ncRNAs (sncRNAs), and long ncRNAs (lncRNAs) (Figure 1). The discovery of rRNA and tRNA dates back to the 1950s, whereas sncRNAs and lncRNAs are relatively recent discoveries [5]. As per our current understanding, molecular size is the distinguishing feature between sncRNAs and lncRNAs [6]. SncRNAs, which have a size <200 nucleotides include microRNA (miRNA), small interfering RNA (siRNA), small nuclear RNA (snRNA), and PIWI-interacting RNAs (piRNAs), whereas the ncRNAs of size >200 nucleotides belong to the category of lncRNAs and are present in almost all the eukaryotic organisms [7].

## 2. Long Non-Coding RNAs (lncRNAs)

**Figure 2:**
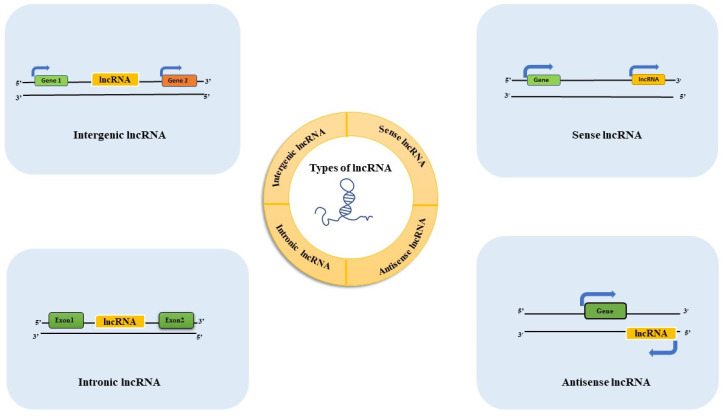
Classification of lncRNAs in the context of genomic locations. Intergenic LncRNAs are transcribed from the genomic region between two coding genes. Sense lncRNA are transcribed from the sense strand of protein-coding genes whereas antisense lncRNAs are transcribed from the opposite strand of coding genes. Intronic lncRNA are transcribed entirely from introns of protein-coding genes.

LncRNAs can be categorized based on the region of the genome from which they are transcribed: (1) Sense lncRNAs overlap the exonic regions of another transcript produced from the same strand; (2) Antisense lncRNAs are located on the complementary strand of the sense strand; (3) Intergenic lncRNAs (lincRNAs) are those produced from the DNA between two genes (Intergenic regions); and (4) bidirectional lncRNAs are transcribed simultaneously to coding transcripts at the opposite strand [9] (Figure 2).

LncRNAs display many common characteristics of mRNAs [10]. The majority of lncRNAs are transcribed by RNA polymerase II and are post-transcriptionally modified [11,12]. They undergo 5′ capping, 3′ polyadenylation, and splicing, but lack poly-A tails [13]. Compared to mRNAs, lncRNAs contain fewer exons, expressed in low abundance and are generally tissue-specific [14]. There are certain databases that are specifically designed for lncRNAs such as NONCODE [15], lncRNAdb [16], lncRNBase [17], DeepBase [18], etc. Even though lncRNAs have been discovered from many insect species, most of our knowledge about the functional aspect of insect lncRNAs comes from studies in *Drosophila melanogaster*. Besides *Drosophila*, lncRNAs have also been reported in *Anopheles gambiae* [19], *Apis mellifera* [20], *Bombyx mori* [21], and *Tribolium castaneum* [22,23]. With the advent of next-generation sequencing (NGS) technologies, large numbers of lncRNAs have been identified, but most of them remain functionallyunvalidated [24]. A total of 11,810 lncRNAs (6250 lincRNAs) were identified in the lepidopteran, *B. mori* [21], which was almost double the lncRNAs identified in many other insect species (3844 lincRNAs from *Plutella xylostella* [25], 2059 lincRNA from *Anopheles gambiae* [19], 3482 lincRNAs from *Aedes aegypti* [26], and 1514 lincRNAs from *Apis mellifera* [20]). This difference could perhaps be attributed to their genome size as well as various annotation strategies, which need bona fide checks. As the lncRNAs are known to regulate several biological processes, viz cell cycle progression, cellular differentiation, development, disease mechanism, metabolism and immune response, understanding the mechanism of action of lncRNAs is very important to regulate the gene expression and epigenetic silencing through heterochromatin formation, histone modulation, and DNA methylation, which are associated with it [27,28,29,30]. Cumulatively, lncRNAs are an important player in regulating gene expressions in several physiological, pathological, and immunological processes [31]. Insect lncRNAs display tight temporal and spatial expression patterns and play important roles in several developmental regulations like sex-determination, immunity, and morphogenesis [32]. They are also known to be involved in determining certain insect behaviors like sleeping, foraging, courtship, etc [33]. A recent study by Stork et al. suggests the existence of 5.5 million insect species, where only about one million species have been characterized to date [34,35]. Furthermore, the least attention has been given to lncRNA studies considering the vast diversity of insects. In this review, we have attempted to summarize lncRNAs and their functions in different insect species studied to date [36].

## 3. Identification and Functional Characterization of lncRNAs

There are at least tens of thousands of lncRNA present in the human genome as well as in the genomes of non-human primates [37]. Similarly, thousands of lncRNAs are also found in other vertebrates, invertebrates, insects, and plants. Furthermore, this number is continuously increasing because of the advancement in sequencing technologies [38]. Since the list is so extensive, no clear orthologous lncRNAs between these groups have been reported. Compared to mRNA, the expression of lncRNA is more tissue specific and its evolution is much faster than mRNA [39]. This is evident by the observation that there is great similarity between the protein-coding genes and miRNA genes of human and mouse, whereas most human lncRNAs do not have any homologs in mice and vice-versa [40]. Interestingly, LncRNAs that are discovered through greater sequencing efforts are less conserved than those previously discovered through shallow sequencing methodologies. Higher sequence conservation exists between exons of lncRNAs compared to that between introns of protein-coding genes [41]. Taking all of the present information about lncRNAs into account, it can be concluded that identifying conserved regions of lncRNAs is a challenging task that would require better prediction tools [42]. For example, it is evident that the evolutionary conservation of long intergenic noncoding RNAs (lincRNAs) might result in the presence of coding chunks and may be biased; as such conserved sequences are significantly reported across all eutherians/mammals [43]. As a result, the functional characterization of lncRNAs and the mechanisms underlying these transcripts lack good precision. Given this paradigm, there is a need for the dissemination of bona fide prediction tools as we hardly know whether insect lncRNAs, if not pest lncRNAs, influence gene expression. Nevertheless, there are reports wherein the transcriptomic changes in plants such as rice take place, but their impact is on the DEGs interacting with lncRNAs alone, suggesting their subtle response to farm chemicals [44]. Altogether, this is also augmented by the fact that insect lncRNAs are mis-annotated by the lack of such annotation tools.

**Figure 3:**
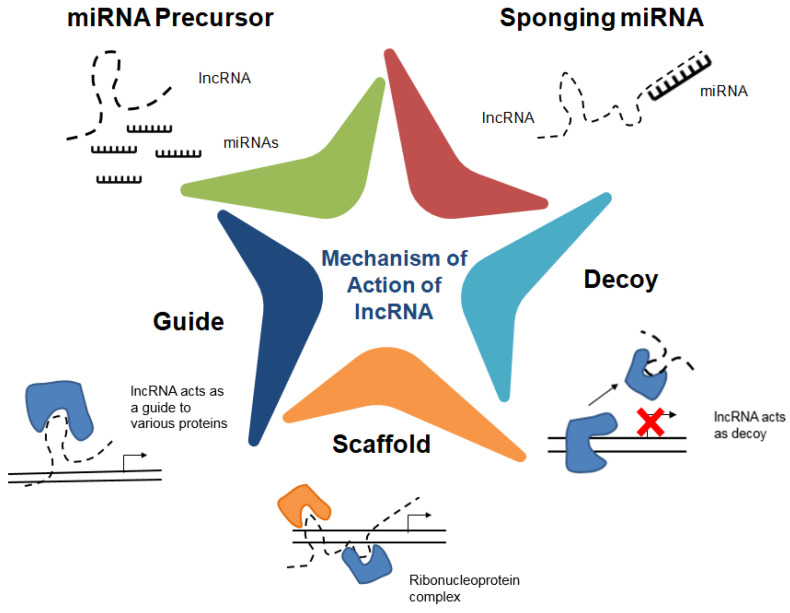
Mechanism of action of lncRNA. LncRNAs regulate gene expressions by acting as guide. They can also act as a scaffold to facilitate the formation of ribonucleoprotein complexes. By acting as a decoy, lncRNA can bind to the transcription factors and remove them from chromatin, thus inhibiting its regulation. LncRNAs can also function as miRNA precursors besides inhibiting miRNA-mediated gene repression by sponging miRNAs.

Since, lncRNAs are relatively less conserved across species, investigating their biological importance as well as their mechanism of action is a difficult task. The discovery of lncRNAs and their functions to date suggest their roles in different biological processes via gene regulation by serving as molecular signals, guides, decoys, and/or scaffolds (Figure 3) [45]. LncRNAs can bind with DNA, RNA, and proteins to regulate gene expressions both at the transcriptional as well as post-transcriptional level. There are different computational approaches for the identification of lncRNAs from the genome as well as transcriptome data, investigation of lncRNA functions, and its associated regulatory networks. Although identifying lncRNAs is out of the question from whole exome sequencing, recent reports including ours have shown how the third generation sequencing technology is used to screen the lncRNAs from exomes [46,47]. Nevertheless, the lncRNAs are ascertained as differentially expressed gene (DEG) counts and many of them turn out to be up/downregulated. These could be from 5′-UTR or intergenic or regulating from the intron-exon boundaries. They are validated using downstream approaches like qRT-PCR or further validation checks. A detailed characterization and potential identification of long read/short-read sequencing were beyond the scope of this review even as potential lncRNAs could be screened from either of these technologies [48].

RNA sequencing is widely used to identify lncRNAs where total RNA is used to generate raw reads, which are then assembled into transcripts either with or without a reference genome and finally, the resulting transcripts are annotated [49]. There are many pipelines available for the identification of lncRNA. Thousands of lncRNAs have recently been identified in *Drosophila melanogaster* using such approaches, which provides a platform for the structural and functional exploration of lncRNA in other invertebrates. Identification of lncRNA started from the Functional ANnoTation Of the Mammalian genome (FANTOM) project, which established a comprehensive platform for the comparative analysis of lncRNAs in mammalian transcriptomes [50]. This was followed by some experimental methods such as tiling arrays and chromatin immunoprecipitation studies. To predict lncRNA–dsDNA interactions, tools like triplextor and Longtarget have been used. LncRNA can be integrated with Hoogsteen hydrogen binding into the main groove of the DNA duplex where Hoogsteen bonding is weaker than the Watson–Crick bonds. Different experimental approaches have been utilized to study lncRNA and DNA interactions. The formation of lncRNA DHFR and dsDNA triplex was demonstrated using electrophoresis mobility shift, and the binding of lncRNA Fendrr to dsDNA was determined by in vitro pull-down experiments [51,52]. Although the mechanism of RNA-dsDNA triplex formation is still not well illustrated, it is clear that the lncRNA–DNA interaction offers a potent mechanism for gene regulation. Similarly, there are different bioinformatic tools to predict RNA–RNA interactions such as RNAplex [53], RNAup [54], and intaRNA [55]. To investigate lncRNA–protein interactions, RPI-Pred [56], lncPRo [57], and RPIseq [58] can be used. There are some tools that can also predict the binding sites of RNAs or proteins such as BindN [59], RNAProB [60], PPRint [61], etc. When the proteins introduced by lncRNAs are methylation-related enzymes, they can induce promoter CpG island methylation or demethylation. If histone-modifying enzymes are imported by lncRNAs, histone changes can lead to gene expression, transcriptional silencing, or repair of DNA and genomic labeling. The neighboring (cis) or distal protein-coding genes (trans) can be regulated by lncRNA [62]. The LncRNAs from one chromatin can bind to another chromatin, like LncRNA HOTAIR, transcribed on chromatin 12 from the HoxC locus and suppresses transcription in the HoxD locus [63]. All these data suggest the complicated roles of lncRNA in gene regulation. In order to gain a deeper insight into the interactions between lncRNA and different biological molecules, extensive experimental validation along with bioinformatics analysis is required. Our understanding of gene regulation by lncRNA is in its infancy, but seeing the large number of lncRNAs studies, we can expect better outcomes in this field in the near future. LncRNAs have been well studied in mammals and are known to be involved in many important biological processes, but it is poorly characterized in non-model organisms such as insects. Excitingly, research focused on insect lncRNAs has increased dramatically in recent years, which suggests their role in insect development, anti-viral defense, and insecticide resistance mechanism. The existing lncRNA studies are limited only to some of the model insects; hence there is a need for the expansion of lncRNA studies in a more diverse range of insects. The discovery and function of lncRNAs in different insects are described below:

### 3.1. LncRNAs in Drosophila Melanogaster

As in the case of other insect specific pathways like sex-determination, metamorphosis, etc., insect model *Drosophila melanogaster* was the first to be attempted for the extensive studies of lncRNA [64,65]. More than 100 lncRNAs are estimated to be coded by the *Drosophila* genome, most of which are expressed during embryonic development and are spatially restricted to the developing central and peripheral nervous system [66,67]. Knockdown studies in *Drosophila* have also confirmed the role of lncRNA in spermatogenesis and male fertility [68]. The function of lncRNAs in *Drosophila* covers development, behavior, stress resistance, gender identification, and dosage compensation [69]. Several testis-specific LncRNAs are required for nuclear condensation and sperm individualization during gonadal development in *Drosophila* [70]. Remodeling of spermatids through chromatin condensation (by replacement of histone proteins with protamines) is essential for the removal of excess cytoplasm required for sperm individualization. Testis of lncRNA (CR44455/6, CR45542, and CR44420) mutant *Drosophila* was found to have spermatids with scattered, round, and un-condensed nuclei [71]. Crumpled nucleus phenotype (similar to that in case of protamine mutants) was also displayed by mutants of lncRNA TS1 and CR43484. Furthermore, RNA sequencing confirmed the role of CR43484 lncRNA in regulating the expression of several testis-specific genes. This included both protein-coding genes and lncRNA, suggesting the involvement of testis-specific lncRNAs in late spermatogenesis through transcriptional regulation (Wen et al., 2016).This is similar to the case of other functionally characterized lncRNAs like Paupar and Pantr [72,73].

A conserved *yellow-achaete* intergenic lncRNA (*yar*) regulates sleep behavior in *Drosophila* [74]. *yar* lncRNA is cytoplasmic and expressed during the mid-embryogenesis [75]. *yar* resides within the neural gene cluster, upstream to the *yar* locus is *yellow* gene (*y*-encodes a secreted protein required for cuticle coloration and male sexual behavior) and downstream is *acheate* gene (*ac*-encodes one of four related bHLH transcription factors of the achaete–scute complex (AS-C) required for proper development of the central and peripheral nervous system [76,77]. There are different lncRNAs that regulate miRNA activities by acting as a sponge to downregulate them. To further investigate the link between *yar* and *Drosophila* miRNAs, sequences encompassing the *yar* exons were submitted to a web-based tool, Microinspector. Using miRBase, which includes both predicted and confirmed miRNAs, 33 miRNA seed matches corresponding to 19 confirmed miRNAs, were identified within *yar* exons. Similar experiments with the *yar* intron resulted in the identification of 36 miRNA seed matches corresponding to 25 confirmed miRNAs. Further studies are needed to investigate the interaction between *yar* and miRNAs and its functional significance. Phenotypic analyses of *Drosophila* null mutants of *yar* suggested its requirement in sleep maintenance and homeostasis. These mutants had reduced night time sleep as a result of reduced sleep bout length. Additionally, there was no increase in the daytime sleep in *yar* null mutants, indicating the loss of sleep homeostasis in these flies and suggesting the involvement of *yar* in sleep regulation. Interestingly, one of the miRNA seed matches within the *yar* exon corresponds to miRNAs from the miR310 cluster (predicted and experimentally confirmed miRNAs). No match was found in similar analyses using intronic sequences of *yar* or exonic sequences corresponding to three other genes (*y*, *ac*, and GAPDH2). Loss of miRNAs 310 to 313 alters synaptic transmission at the larval neuromuscular junction, with no effect on viability or fertility. These findings are consistent with the possibility that *yar* might participate in a regulatory circuit that influences levels of miRs within the brain, which may have the capacity to contribute to synaptic homeostasis [74]. Comparative sequence analysis of the *yar* locus (with one of the conserved sequence motifs bound to the *yar* promoter) suggested its conservation in different *Drosophila* species, representing 40–60 million years of co-evolution. The similar timing of *yar* expression in *D. virilis* and *D. melanogaster* further suggests conservation in the transcriptional regulation of *yar* [78].

Nuclear lncRNAs, roX1 and roX2, are essential for the process of dosage compensation in *Drosophila* [79]. Both of these lncRNAs (roX1 and roX2) form the male-specific lethal (MSL) ribonucleoprotein complexes. It was observed that synchronous removal of lncRNA-rox1 and roX2 decreases X-chromosome localization of the MSL complex. Interestingly, both these lncRNAs independently play an important role in the dosage compensation process where roX1 is the most abundant one and its loss results in the reduced expression of X-chromosome genes. Loss of roX2 leads to MSL independent upregulation of genes [80,81]. Similarly, Xist is a lncRNA involved in dosage compensation in humans where it regulates chromatin modification and expression of specific genes. *Sexlethal* (*Sxl*) is the master regulator of the sex-determination process in *Drosophila* [82]. The dose sensitive early promoter of *Sxl* (i.e., *SxlPe*) senses the number of X chromosomes (one X vs. two X) and gets transcribed only in females (i.e., with XX sex chromosome composition) [83]. Since sex determination and dosage compensation processes are linked and lncRNAs are involved in the dosage compensation process in *Drosophila*, researchers have also speculated on the role of lncRNAs in the activation of *SxlPe* [84]. *Drosophila* lncRNAs, heat-shock RNA-omega (hsr-omega/ω), and CR34335 are involved in the process of cellular aging [85]. hsr-ω, present in the form of nucleoplasmic omega speckles, is essential for accumulating heterogeneous nuclear RNA binding proteins (hnRNPs) [86]. hsr-ω functions as a hub for the coordination of transcriptional regulators and hnRNPs, which impact many cellular responses such as apoptosis [87]. The 93D, or hsr- ω (heat-shock RNA-omega), locus of *Drosophila* is known to be involved in transcriptional and translational activities. Initially, this gene was named 93D as it is present in the 93D cytogenic region of the polytene chromosome of *D. melanogaster*, but was later renamed as hsr-ω (heat shock RNA-omega). This gene was compared in three different *Drosophila* species: *D. hydei*, *D. melanogaster,* and *D. pseudoobscura*. Although the structure of this locus is highly conserved in all three *Drosophila* species, the primary base sequence had diverged rapidly between them. In all three species, hsr-ω consists of a unique region in the transcription unit at 5′ site, which consists of two exons and one intron [88]. Overall, three transcripts (two nuclear and one cytoplasmic) are produced from this locus, which do not have any significant protein-coding capacity. This locus is developmentally active in nearly all cell types and is essential for the viability of flies. Its induction during heat shock is independent of the other members of the heat shock gene family [89]. The other selective inducers act on this locus through separate response elements and hsr-ω activity has a characteristic effect on transcription/turnover of the heat shock induced hsp70 and the alpha-beta transcripts in *Drosophila melanogaster* [90]. Research has shown that lncRNA-hsr performs a crucial function in thermo tolerance to cope with heat stress [91]. Upon temperature shock, the nullisomy, RNAi, or overexpression of lncRNA-hsrω imply lethality in most embryos and first or third-instar larvae [92]. Three-day-old null fly lncRNA-hsr assemblies have poor projections after heat shock, whereas both down- and upregulation of lncRNA-hsr assemblies reappear during the recovery from heat shock [93,94].

The *Drosophila* maternal effect gene oskar encodes the protein oskar and has distinct roles in germ line determination and posterior abdominal segment differentiation [95]. There are two isoforms of the Oskar protein, which are formed as a result of translation of two different in-frame start codons of *oskar* mRNA. Out of these two, the short oskar (139–606aa) is absolutely required for germ cell development whereas the long oskar (1–606aa) is essential for tight anchoring of the germplasm to the posterior oocyte cortex. Nonetheless, oskar RNA plays a major role during early *Drosophila* oogenesis via a translation-independent mode that acts as lncRNAs [96]. Most oskar RNA levels have been decreased by a sterile phenotype due to early oogenesis. Moreover, expression of the oskar 3′ UTR is sufficient to recover the eggless defect of the RNA null mutant independent of protein [96]. Previously, the localization of Staufen, an RNA-binding protein, within the oocyte is interdependent with that of *oskar* mRNA [97]. In the *oskar* null mutant, the Staufen protein fails to transport from the nurse cells into the oocyte. Expression of the *oskar* 3′ UTR alone is sufficient to restore Staufen accumulation in the oocyte. This reveals that the mutual interdependence of Staufen and *oskar* RNA in their localization during oogenesis is mediated by the interaction of Staufen with the *oskar* 3′ UTR [98]. Another possibility is that this non-coding function is mediated partly through sequestration of the translational regulator Bruno, which binds to Bruno response elements in its 3′ UTR [99]. Another important lncRNA in *Drosophilamelanogaster* is acal, which is involved in negative regulation of Jun-N-terminal kinase (JNK) signaling and maintaining cell stretching and closure [100]. Bereft and bxd, other important lncRNAs, are involved in the development of extra sensory organs [101] and are also involved in growth and development, respectively [102,103]. Iab-8 represses the homeotic gene abd-A and its knockdown leads to male and female sterility [104]. Another important lncRNA in *D. melanogaster* is CRG, which is involved in locomotor activity and climbing ability [105]. There is a controlled expression of lncRNA-CRG in the central nervous system (CNS) from the embryonic to the adult stages. This lncRNA shows high sequence conservation across the 12 *Drosophila* species. LncRNA-CRG is located downstream of the Ca2+/calmodulin-dependent protein kinase (CASK) and partially overlaps with the 30 UTR of CASK, where CASK is a behavior-related coding gene. All these lncRNAs and their properties are summarized in Table 1.

### 3.2. LncRNAs in Apis Mellifera

Honey bees have socio-economic importance for both mankind and nature. The division of labor in honey bees has been well studied, which represents an excellent coordinated work of society [106]. Worker bees have an age-dependent division of labor; young worker bees perform the duties of nursing the brood and old ones are involved in foraging (collection of nectar and pollen) [107]. This transition is thought to be regulated by the interaction of yolk protein, vitellogenin (Vg), endocrine factor, juvenile hormone, and the biogenic amine, octopamine. Furthermore, microarray analysis revealed the preferential expression of certain genes (foraging, malvolio, Hormone-like Receptor in 38 (HR38)) in the forager bee brain. LncRNAs have been found to function in developmental processes in honey bees [108]. Enhanced expression of a non-coding RNA, *Nb-1*(*Nurse bee brain-selective gene-1*) was found in the brain of nurse bees compared to that in forager and queen bee brains in normal colonies [109]. *Nb-1* is specific only to some hymenopteran species. Nb-1 is involved in age-dependent transition in worker bees by regulating the synthesis and secretion of octopamine and juvenile hormones [109]. Other lncRNAs reported in the brain of honey bees are KS-1, AncR-1, and kakusei. KS-1 (Kenyon cell/small- type preferential gene-1) is a 17 kb nuclear transcript that is preferentially expressed in Kenyon cells of the honey bee brain [110]. Kenyon cells are subtypes of inter neurons in mushroom bodies (MB), which is an essential and regulatory part of the insect brain. *Ks-1* positive neurons were found to be greater in drones than in queens between the lateral calyx and the optic lobes of the brain, suggesting their involvement in drone specific brain functions. Overall nucleotide sequence of *Ks-1* was found to be conserved among the honey bees [111]. AncR-1 is another lncRNA that was discovered in honey bee brains and is also expressed in sex and secretory organs. Localization of AncR-1 and Ks-1 transcripts in a distinct portion of a single neural nucleus suggests their involvement in distinct neuronal functions in the brain [112]. The kakusei locus produces both constitutive- and inducible-type variants of the kakusei transcript in the brain of worker bees [113]. Some kakusei transcripts are produced as a result of increased neural activity whereas some are constitutively expressed (i.e., independent of neural activities). Since, there is neither an alternative splicing (within the kakusei locus) nor extended transcription from the kakusei cDNA sequence, constitutive-type kakusei variants might be produced by differential transcription initiation or termination within the anterior part of the kakusei cDNA sequence. Both kinds of transcripts are localized predominantly in the neural nuclei and might be related to different nuclear functions [113,114]. LncRNAs, lncov1 and lncov2, are intronic lncRNAs (located within the first introns of their host genes) that are expressed in honey bee ovaries. The host gene for lncov1 is ‘LOC726407′ (with unknown function) and lncov2 is fringe, a homolog of an important *Drosophila* developmental gene, with the latter, found to be over-expressed in larval queen ovaries and was involved in JH-dependent maintenance of developing ovarioles in the early fifth instar of queen larvae [115]. The expression of lncov1 was found to be maximum in the fifth instar larvae, exactly before they enter into metamorphosis. This coincides with the autophagic cell death in the larval worker ovary, which indicates the role of lncov1 in autophagic cell death during larval to pupal metamorphosis.

Apart from various species of honey bees, two significant species—*Apis mellifera* (Western honey bee) and *Apis cerana* (Asian honey bee)—play crucial roles in development, social behavior, and disease transmission, and also serve as the significant pollinators of economically important crops [125,126]. A total of 2470 lincRNAs, encoded by 2376 gene loci in the *A. cerana* genome and a total of 1514 lincRNAs in the *A. mellifera* genome were identified in silico [20]. These lincRNAs were presumed to be associated with functions like hormone signaling, metabolism, association with diseases, and role in gene modulation. The comparative analysis of lincRNA between two sister species resulted in high conservation among them, and only 5% were found to be unique to each species. Both species showed tissue-specific expression of lncRNAs; in the case of *A. cerana*, more lincRNA were expressed in the fat body and antenna while in *A. mellifera*, expression was visible in the ovary and brain [20]. These results were considered to be the proponent of the role of lincRNA in the major metabolic and hormone signaling pathways in insects. Chen et al. performed strand specific whole transcriptome RNA-sequencing of control and *Nosema ceranae* infected midgut samples of *Apis mellifera ligustica* workers. This study resulted in the identification of a total of 6353 lncRNAs out of which 4749 were conserved lncRNAs and 1604 were novel lncRNAs. Interestingly, these lncRNAs did not show any similarities with other known lncRNAs in other species, though there was some structural similarity with counterparts in mammals and plants. Additionally, 27 discovered lncRNAs were harboring eight known miRNA precursors. This study gave a basis for understanding the host–pathogen interaction in *Apis mellifera* and investigating the roles of lncRNAs associated with this process [127].

### 3.3. LncRNAs in Aedes Aegypti

*Aedes aegypti* (the yellow fever mosquito) is a major vector of arboviruses including dengue, Zika, and Chikungunya viruses [128,129]. A recent study in *Aedes aegypti* has led to the identification of 4689 novel lncRNA transcripts, which includes 2064 intergenic, 2076 intronic, and 549 antisense lncRNAs [130]. Genome-wide analysis and developmental profiling of these newly identified lncRNAs suggests that a subset of lncRNAs shows maternal inheritance and early embryonic expression [130]. One of the probable reasons for implicating the involvement of lncRNA in developmental regulations is their proximal location to the genes involved in various developmental processes. High expression of few lncRNAs was also found in the ovary of blood-fed mosquitoes, which lasts up to 12 h of embryonic development, suggesting their maternal supply [131]. The temporal expression and maternal inheritance of lncRNA in *A. aegypti* account for developmental transition in mosquitoes [132].

### 3.4. LncRNAs in Anopheles Gambiae

Jenkins et al. identified 2949 lncRNAs from different life stages of malaria mosquito *Anopheles gambiae* using RNA-Seq data [18]. These authors observed that most (2059) of the identified lncRNAs were intergenic, whereas some (108) were anti sense and some (782) were mapped within the intronic region of protein-coding genes [18]. These lncRNAs displayed very little sequence conservation compared to protein-coding genes in different *Anopheles* species. Interestingly, the secondary structural features for many lncRNAs were found to be conserved. While the lncRNAs are thought to be the potential targets of epigenetic regulation and controlling vector-transmitted infectious diseases, they are also known to impede the ncRNA targets in vector insects. The evolution of lncRNA secondary structures tends to follow the concept of ‘pervasive transcription’ (i.e., most regions of the genome are transcribed, even those that failed to encode proteins or functional ncRNAs) [133].

### 3.5. LncRNAs in Bombyx Mori

Domesticated silkworm, *Bombyx mori* is an economically important insect [134]. Besides being the primary producer of silk, it is also a lepidopteran model insect that has attracted researchers in answering several biological questions. Silkworm displays several sexually dimorphic behaviors that are associated with the insect brain. A lncRNA, Fben-1(female brain expressed noncoding RNA-1)is preferentially expressed in the cells surrounding the mushroom bodies in the brain of female silkworm, suggesting the involvement of Fben-1 in certain neural or developmental/sexual functions in females [135]. *Fben-1* is located ~6 kb upstream of the *fruitless* (*fru*) gene in the *B. mori* genome. LncRNA Bmdsx-AS1is expressed abundantly in *B. mori* testis and is involved in the sex-specific alternative splicing of pre-mRNA of its *doublesex* (*Bmdsx*) gene [136]. *doublesex* (*dsx*) has been found to be conserved in all the insects studied to date [137]. The pre-mRNA of *dsx* is alternatively spliced to produce sex-specific transcripts, which ultimately make sex-specific proteins responsible for all kinds of sexual dimorphism in insects [138].

*Bmdsx-AS1 lncRNA* interacts with BmPSI (*B. mori* P element somatic inhibitor) via a splicing factor hnRNPH [118]. BmPSI is a male-specific protein that promotes male-specific splicing of *Bmdsx* pre-mRNA by binding to the CE1 sequence (exonic splicing silencer) present in the fourth exon of *Bmdsx*, leading to its skipping [139]. Another lncRNA of *B. mori*, iab-1, is coded by the intergenic region between Bmabd-A and Bmabd-B in the Homeobox (Hox) cluster of the silkworm. At least seven alternatively spliced iab-1 lncRNAs are produced and are expressed at specific developmental stages. The siRNA mediated downregulation of iab-1 leads to the death of larvae, suggesting its involvement in certain essential physiological processes [119]. A recent study showed the expression of lncRNAs in response to infections. *B. mori* nucleo-polyhedrovirus (BmNPV) is threatening to economic growth and the stability of sericulture business. The BmNPV infection in silkworms leads to the expression of 4450 lncRNA [140]. In-silico analysis suggests the target of these differentially expressed lncRNAs to be the genes involved in ubiquitin mediated proteolysis, endocytosis, and lysosomal pathways [141].

### 3.6. LncRNAs in Plutella Xylostella

Researchers have identified 3844 lincRNA from *Plutella xylostella* (a deadly pest of cruciferous plants), which has developed field resistance to all classes of insecticides. Some of the identified lincRNAs were found to serve as the precursor of small ncRNAs. These lincRNAs can be linked to the development of resistance to a large variety of insecticides in *P. xylostella* as differential expression of lincRNA was observed in the larvae of *P. xylostella* that were resistant to insecticides like organophosphate, phenylpyrazole, and Bt endotoxins [24,142]. Interestingly, most of the presumptive lincRNAs were found to be over expressed whereas some were found to be repressed in deltamethrin resistant larvae. Previous studies have shown the differential expression of lncRNAs with respect to various stress factors. Recently, a study compared the dsRNA induced lncRNAs in three insect species: *Helicoverpa armigera, Plutella xylostella*, and *Tribolium castaneum.* A total of 3463 *H. armigera*, 6245 *P. xylostella*, and 3067 *T. castaneum* differentially expressed lncRNAs were identified with respect to dsRNA induction [121].

### 3.7. LncRNA in Planthopper Insects

Well-known crop pests of Asia include *Soagetlla furcifera* (white-backed planthopper, WBPH), *Nilaparvata lugens* (brown planthopper, BPH), and *Laodelphax striatellus* (small brown planthopper, SBPH). With the help of NGS, genomes of all three planthoppers were sequenced, resulting in the identification of many functional genes [143,144,145]. From this study, 2439 lncRNA transcripts were reported in BPH [146], 5790 lncRNA transcripts were identified in SBPH [147], and 1852 lncRNA transcripts were found in WBPH [148]. Interestingly, these lncRNAs from different insects shared similar sequences due to their close genetic relationships. These lncRNAs were found to play distinct roles in fecundity, virulence, and developmental regulation.

## 4. Challenges and Future Perspectives

During the past few years, studies on lncRNAs have steadfastly gained momentum, albeit the insect group remains unexplored. Despite the large diversity of lncRNAs, the study of lncRNA is either limited to few model insects or is at the elementary level of research in other insects. Given the fact that the lncRNAs possess a greater potential to interact with other genetic materials like DNA, RNA, or protein molecules, it is likely that gene expression studies would yield possible insights into understanding their biological phenomena. However, due to the scarce information about the functional aspects of lncRNAs, there is a need to resolve the notion of the hidden non-coding potential of lncRNA. In the past decade, many technologies and strategies were tailored to identify and characterize lncRNAs. Continual efforts in the direction of annotating different kinds of lncRNA has allowed for the discovery of their biological roles, although the significant challenges of filtering out ncRNAs from coding RNA and the lack of proper bioinformatics tools to identify multifunctional RNA still exist. The development of highly sensitive and specific techniques is required to identify interactions between lncRNAs and other molecules (chromatin, RNA, protein) or locating the molecules found in proximity to lncRNA in order to determine their regulatory functions. The insufficient knowledge and limited potential of in silico tools to predict the mechanisms and functional domains within an lncRNA also poses a major task. Previously, it was considered to be the junk part of the genome, but later came to light as a key regulator in chromatin remodeling, DNA methylation, and RNA editing. Apart from epigenetic regulation and chromatin remodeling, lncRNAs are also known to serve developmental processes, immunity responses, etc. Hence, lncRNAs can potentially be used in therapeutics. For example, in *Drosophila*, few lncRNA perform a vital role in neurogenesis and spermatogenesis; this point marks their importance in countering any fetal abnormalities [66,71]. The involvement of lncRNA in cellular processes like oncogenesis and tumor suppression may provide an opportunity of developing lncRNA as therapeutic targets of cancer [149]. Likewise, in mammals, lncRNAs exhibit varying expression levels upon severe acute respiratory syndrome coronavirus (SARS-CoV) viral infection, regulating the innate immune response [150]. Similarly Zhou et al. also accounted for evidence regarding the association of Arid2-IR lncRNA with renal fibrosis and renal inflammation [151]. One particular importance of lncRNA is their involvement in host–virus interaction and conferring insecticide resistance in insects [28,152]. Due to the significant heterogeneity of lncRNAs, they can be potentially used in improving insect control strategies as well as in predicting the mechanisms underlying the developmental processes as they are commonly designated as time-specific tuners by governing the time of developmental transitions.

## 5. Conclusions

The complex patterns of expression and regulation of the entire eukaryotic genome are in the hands of ncRNAs. The ncRNAs participate in almost all sorts of biological processes including epigenetic control of traits, transcriptional, and post-transcriptional regulations. Interestingly, a growing number of ncRNAs have been identified in insects primarily from the RNA-Seq of RNA libraries or transcriptomics studies. Small ncRNAs such as miRNAs and piRNAs have been well studied compared to lncRNAs. In this review, we discussed the importance of lncRNAs in different insect orders. Keeping in mind the rationale that insects are the most abundant and diverse group of animals and play an important role in different ecosystems aside from significantly contributing to the population dynamics, their influence on the ecological and economic environments of humans is undebated. Studies on RNAs and their role in gene regulation using insect models will open windows to understanding the immune response and disease dynamics. Although there are currently many studies on lncRNAs in insects, all these studies are still at the preliminary stages and focus solely on model insect species. One particular aspect, which could be promising, is to compare RNA at the cross-sectional level. Furthermore, insects are particularly ectothermic and global warming has pushed them to newer territories where human interaction has increased many fold. In this scenario, ncRNAs, particularly lncRNAs, are a promising solution to novel diseases. An essential aim of developing our understanding of lncRNAs is to create environmentally-friendly and efficient pest control strategies for agricultural pests. Therefore, lncRNAs have great potential to be used as targets in pest control strategies in the future.

## Figures and Tables

**Figure 4:**
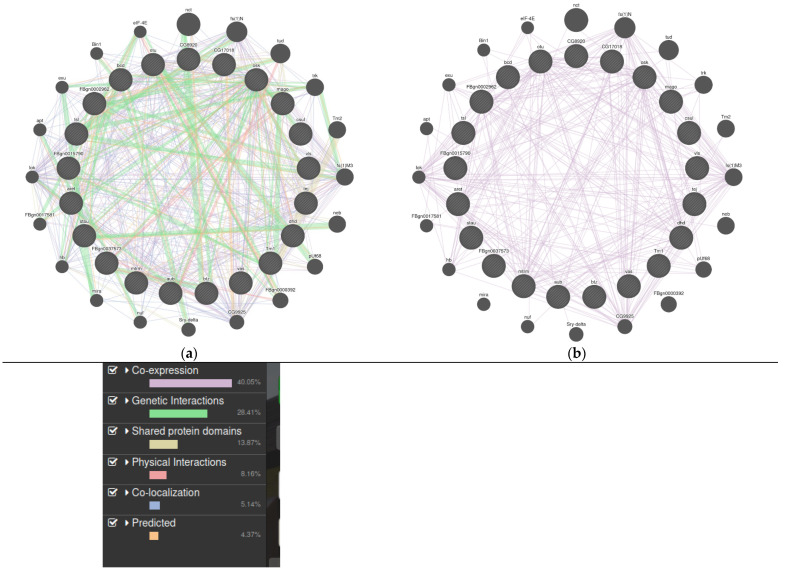
(**a**) A detailed protein–lncRNA interaction map generated using GeneMania that shows physical interactions, co-expression, co-localization, pathways, and genetic interactions in different edge colors (Supplementary file) The genes were queried using GeneMania with *Drosophila melanogaster* as a query producing the candidate nodes shown to be interacting with others. (**b**) A co-expression interaction network of lncRNAs in insects. The edges (in pink color) indicate the co-expression between these lncRNAs and their interacting partners. It is likely that all co-expressed interactants are co-localized. Approximately 40.05% of interactions were associated with co-expression (please see legend below the figure).

**Table 1:** Table summarizing the important lncRNAs in different insect orders (Figure 4).

S.No.	Organism	Name of lncRNA	Function	Size (bp)	Site of Expression	Interaction with Other RNA/Protein	Reference
	Order: Hymenoptera						
1	*Apis mellifera*	Nb-1 (Nurse bee brain-selective gene-1)	Shows varying expression in the brain of honey bees relative to the age of the colony.	599	Brain	Octopamine and Juvenile hormone	[109]
2		KS-1 (Kenyon cell/small-type preferential gene-1)	Expresses in the mushroom body of Kenyon cells in the honey bee brain and is accumulated in the nucleus. It is also involved in neuronal functions.	17,525	Kenyon cells	-	[110,112]
3		AncR-1	Exhibits spatial expression in the brain, sexual tissues, and in some secretory organs. It is also involved in neuronal functions.	6861	Sexual tissues and secretory organs	-	[112]
4		Lncov1 and Lncov2	Shows differential expression in the ovarioles of queen and workers.	1367	Larval Ovary	[116]	[110]
		Order: Lepidoptera					
5	*Bombyx mori*	dw4sg_0040	Downregulates the antibacterial peptide, metabolic process, and oxidative response.		Silk gland	-	[117]
6		dw4sg_0178	It is downregulated in *B. mori*		-	-	
7		dw4sg_0483	Performs the post-transcriptional regulation of silk protein to yield silk		-	-	
8		Bmdsx-AS1	Promotes male specific splicing of Bmdsx by interacting with BmPSI		Silkworm testis	Hnrnph, BxRBP1 &3.	[118]
9		iab-1	Involved in essential metabolic/physiological processes.	~>1000	Nervus and epidermis	Interacts with Hox gene- BmUbx,, Bmabd-A, Bmabd-B	[119]
10		Fben-1	Developmental/sexual functions in females.	~2000	Female brain (mushroom body)	-	[120]
11	*Plutella xylostella*	TCONS_00186426	Co-expresses with glycoprotein, Abd-5, which is important for cuticle formation in insects.	-	-	Abd-5	[121]
12		TCONS_0002929	Indirectly involved in fruit fly development as it expresses nearby C-roughest protein rst, which has a role in the development of fruit fly	-	-	-	
13		TCONS_00008658	Located in an intergenic region near JHEJ. JHEJ is known to activate insect JH.	-	-	-	
		Order: Diptera					
14	*Drosophila melanogaster*	roX1 & roX2	Regulates dosage compensation and formation of MSL ribonucleoprotein complex.	4832 & 1368 respectively	roX1 expressed in Nuclei of all body parts. roX1 and roX2 expressed in the CNS of male brain.	Interacts with roX1: Clamp, Unr, mle, mof, MSL2,3. roX2: Male less (MLE), Male Sex Lethal (MSL1,2,3), mof, Unr.	[81,122]
15		hsrw	Regulates the development of neuromuscular junctions.	21,216	Expresses in almost every stage.	Hrb87F, Hrb98DE, Iswi, Mtor, Pep, Saf-B, TBPH, caz, sqd, hrp-40.	[89,94,123]
16		yar	Regulates the sleep behavior and the circadian rhythm.	1569	Embryo and cytoplasm	-	[74]
17		Sphinx	Directs male courtship behavior mediated by olfactory neurons.	644,454	-	*Or92a.*	[124]
18		acal	Involved in sealing the dorsal gap during embryonic development.	2386	Embryonic/larval: CNS, epidermis	aop, bsk, raw	[100]
19		oskar	Works along with Staufen to regulate oogenesis.	2335	Germplasm	Stau, vls, nos, SmD3, bwk, etc.	[96]
20		bereft	Role in development of extra sensory organs (interommatidial bristles).	~7000	Peripheral nervous system, non -neuronal epidermis.	Krn, Nach, hid, ppk16, ppk28, ppk6, spi, upd1-3, vn	[101]
21		msa	Responsible for male fertility and development of accessory glands.	>6500	Secondary cells of drosophila male accessory glands	-	[68]
22		bxd	Associated with repression of ultrabithorax (ubx) and also regulates growth and development.	1755	-	-	[103]
22		iab-8	Inhibits expression of homeotic gene abd-A. Knockdown of iab-8 causes sterility in both sexes.	92,000	-	-	[68,104]
23		SxlPe-R1 and R2	Facilitates sex determination.	480	-	-	[83]

## Data Availability

Not applicable.
